# Food Insecurity in a Pediatric Emergency Department and the Feasibility of Universal Screening

**DOI:** 10.5811/westjem.2021.7.52519

**Published:** 2021-10-27

**Authors:** Jaqueline Valdez Gonzalez, Emily A Hartford, Jennifer Moore, Julie C. Brown

**Affiliations:** * Seattle Children’s Research Institute, Emergency Department Research Team, Seattle, Washington; † University of Washington, Department of Pediatrics, Division of Emergency Medicine, Seattle, Washington; ‡ University of Washington, Department of Pediatrics, Seattle, Washington

## Abstract

**Introduction:**

Children with food insecurity (FI) experience adverse health outcomes due to inadequate quantity or quality of food. Food insecurity may be high among families seeking emergency care. The Hunger Vital Sign (HVS) is a two-question validated tool used to screen families for FI. Our goal in this study was to assess prevalence of FI among emergency department (ED) patients, patient-level risk factors for FI, and the feasibility of screening.

**Methods:**

This was a cross-sectional analysis of FI in the ED. Parents or guardians of ED patients and adult patients (18 years or older) were approached for screening using the HVS during screening periods spanning weekdays/weekends and days/evenings. All ED patients were eligible, excluding siblings, repeat visits, critically ill patients, minors without a guardian, and families that healthcare staff asked us not to disturb. Families answered the HVS questions verbally or in writing, based on preference. Families with positive screens received information about food resources. We summarized patient and visit characteristics and defined medical complexity using a published algorithm. Multivariable logistic regression was used to assess FI risk factors.

**Results:**

In July–August 2019, 527 patients presented during screening periods: 439 agreed to screening, 18 declined, 19 met exclusions, and 51 were missed. On average the screening tool required five minutes (range 3–10 minutes) to complete. Most families (328; 75%) preferred to answer in writing rather than verbally. Overall, 77 participants (17.5%) screened positive for FI. In regression analyses, FI was associated with self-reported race/ethnicity (combined variable) of African American or Black (odds ratio [OR] 5.21, 95% confidence interval [CI], 2.13–12.77), Hispanic (OR 3.47, 95% CI, 1.48–8.15), or mixed/other (OR 3.81, 95% CI, 1.54–9.39), compared to non-Hispanic white. FI was also associated with public insurance type (OR 5.74, 95% CI, 2.52–13.07, reference: private insurance), and each year of increasing patient age (OR 1.05, 95% CI, 1.01–1.09). There were no associations between FI and medical complexity or preferred language.

**Conclusion:**

Food insecurity was common among our ED patients. Race and ethnicity, insurance status, and increasing patient age were associated with increased odds of FI. Efforts to include universal FI screening for ED patients with immediate connection to resources will enhance overall care quality and address important health needs.

## INTRODUCTION

Food insecurity (FI) is the limited or uncertain availability of nutritionally adequate foods, or limited ability to acquire such foods in socially acceptable ways.^1^ Food insecurity is a critical determinant of child health and is associated with worse healthcare access and poor health outcomes.^2^–^5^ It has been linked to a variety of conditions including developmental delay, behavioral dysregulation, poor academic school performance, asthma, depression, and anxiety.^6^ Children living in homes with FI have more frequent viral infections, chronic medical conditions, and lower levels of psychosocial and physical functioning.^2^,^7^–^9^ In addition, stress produced by ongoing food insecurity may predispose children to other chronic diseases such as diabetes, hypertension and obesity, with effects that continue into adulthood.^4^,^10^

Food insecurity is increasingly common in the United States (US), involving approximately 13.6% of US households with children.^4^ It appears to be more prevalent in families presenting to the pediatric emergency department (ED) than the general population, with reported prevalence between 20–46%.^11^–^14^ Children in food-insecure households may use the ED more frequently; therefore, this clinical setting presents opportunities for identifying needs and making connections to food resources. The prevalence of and risk factors for FI among patients in the ED in our region have not been well established.

The Hunger Vital Sign tool (HVS) is a validated, two-question screening instrument that is highly sensitive and specific for FI.^15^ The HVS identifies households as being at risk for FI if answers to either of the following statements are “sometimes true” or “often true”: 1) *Within the past 12 months we worried whether our food would run out before we got money to buy more;* or 2) *Within the past 12 months the food we bought just didn’t last and we didn’t have money to get more*.^15^ The HVS is recommended for use by the American Academy of Pediatrics for universal screening for FI during routine visits with children.^16^

Our aim in this study was to assess the prevalence of FI using the HVS among patients visiting our academic, freestanding pediatric ED, the feasibility of screening, and the demographic associations with FI in our population.

## METHODS

This was a cross-sectional analysis of the baseline prevalence and risk factors for FI and an assessment of the operational feasibility of screening in our ED. We utilized the STROBE checklist for cross-sectional studies (Supplement). A convenience sample of families and adult patients presenting to the ED were approached during screening blocks across a range of weekday and weekend days. The screening blocks were 3–4 hours long, covering the range from 8 am to 10 pm and included coverage seven days per week. Approximately one quarter of screening blocks occurred on weekend days and the remainder throughout the week. Families were screened for FI using the HVS.

All families arriving to the ED within screening hours were eligible to be approached for the study. We excluded siblings, repeat visits, critically ill patients, minor-age patients without a guardian, and families that clinicians asked us not to disturb. In most cases an adult caregiver for the patient was asked to answer the screening questions. If the patient was an adult (18 or older) and no adult caregiver was present, the patient was asked directly. The respondent answered two FI screening questions verbally or in writing, based on preference. The written screening questions were offered in Spanish and Somali in addition to English, as these are the three most spoken languages in our ED.

**Population Health Research Capsule**

What do we already know about this issue?

*Children with food insecurity (FI) experience adverse health outcomes. The prevalence in the United States is approximately 13.6%.*

What was the research question?

*What is the prevalence of and risk factors for FI in our emergency department, and is universal screening feasible?*

What was the major finding of the study?

*In our emergency department (ED), FI was 18% with these risk factors: Black and Hispanic race/ethnicity; increasing age, and non-private insurance. Screening took 5 minutes to complete.*

How does this improve population health?

*Our findings provide urgency and insight to implement universal screening for food insecurity in pediatric EDs to address inequities in health outcomes for children.*

All other languages comprise a small proportion (<3% each) of our patient population. For patients who expressed a preference for care in another language, questions were asked verbally using an interpreter. Those who screened positive received information about food-related resources in the community and resources specific to our hospital including an onsite food pantry. This information was provided through handouts that were available in English and Spanish. Families with a language of care that was not English or Spanish received information about the food-related resources using a telephone interpreter. Families were also offered a visit with an ED social worker to address any other needs they might have. Clinicians were informed if their patient screened positive for FI.

We summarized patient characteristics using descriptive statistics. Continuous variables were assessed for normality and, if normally distributed means and standard deviations. If not normally distributed, medians and interquartile ranges were used. Categorical variables were summarized using frequencies and percentages. We reported race and ethnicity using a combined race/ethnicity variable using an approach that has been discussed in the literature.^17^ The patient’s race and ethnicity were self-reported separately; they were categorized as Hispanic if they identified as Hispanic ethnicity, including any race. For non-Hispanic ethnicity, race categories were separately reported.

We included patient complexity level using the patient medical complexity algorithm (PMCA), which uses billing and diagnosis data to stratify children based on presence of chronic and/or complex disease.^18^ The patient’s preferred language was determined based on parent report during registration of what language they would prefer for care during their visit. High- and low-volume hours were classified based on historical ED encounter data; ED visits between 2 pm–2 am were considered higher volume hours and between 2 am – 2 pm as lower volume. We used multivariable logistic regression to assess risk factors for FI. Results were reported as odds ratios (OR) and 95% confidence intervals (CI). A *P*-value of 0.05 was considered statistically significant. SAS 9.4 (SAS Institute Inc, Cary, NC) was used for all analyses.

We based feasibility on the time required to screen and to provide real-time resources for patients who screened positive. This study was granted exempt status by the hospital’s institutional review board.

## RESULTS

There were 527 pediatric ED patient encounters eligible within the screening hours. Of these, 457 patient caregivers or adult patients were approached and 439 (96%) agreed to participate in screening and were screened; 18 declined, 19 met exclusion criteria, and 51 were missed (Figure). On average, the FI questions using the screening tool required five minutes (3–10 minutes) to complete; the screening required closer to 10 minutes when an interpreter was used. The majority of participants (328; 75%) preferred to answer in writing rather than verbally. Overall, 77 participants (17.5%) screened positive for FI (Table 1).

In our regression model, several patient factors were associated with higher odds of FI (Table 2). Patients and families were more likely to have food insecurity if they self-reported their race/ethnicity to be Black (OR 5.21, 95% CI, 2.13–12.77), Hispanic (OR 3.47, 95% CI, 1.48–8.15), or Mixed/Other (OR 3.81, 95% CI, 1.54–9.93) when compared to non-Hispanic white. Families with public insurance were more likely to report food insecurity than those with private insurance (OR 5.74, 95% CI, 2.52–13.07). Each year of increasing patient age was associated with a 5% increased odds of FI (OR 1.05, 95% CI, 1.01–1.09). There was no association between FI and presence of chronic conditions using the PMCA. There was also no statistically significant association with preferred language of English or non-English.

Families that screened positive were provided with information about additional resources at Seattle Children’s and within the community. Providing this information required an additional 10–15 minutes depending on the family’s number of questions, need for interpretation, and interest in engaging in more conversation or requests for additional resources. Additional time was also needed to maintain accurate and updated resources for families, which were also translated into Spanish.

## DISCUSSION

The prevalence of FI in this sampling of our ED population was 17.5%, exceeding what has been reported in households with children nationally. It is slightly below what has been reported in EDs in other US cities, with variability by region.^11^–^13^ Our patient population is diverse and unique because our hospital both cares for patients from the nearby major urban center while also functioning as the main subspecialty referral center for a large region including five states. In Philadelphia, 20.6% of 1,818 participants screened positive for FI using the HVS.^11^ In Maryland, among patients under four years of age, 22.7% of 3800 participants screened positive for FI based on the 18-item Household Food Security Survey Module and 32.9% using the HVS.^12^ In Madison, Wisconsin, 45.6% of 309 caregivers screened positive using the HVS and non-White race/ethnicity was associated with higher FI (56.8% vs 27.4%, *P* <0.01).^13^

In our study, we also found there was a significantly higher risk of screening positive for FI among those who identify as Black or Hispanic. This finding is in line with a large body of literature on structural racism and its many ill effects on communities that have been historically marginalized.^19^, ^20^ Families who identify as Black or Hispanic are more likely to be experiencing FI when they arrive in our ED. Raising awareness of this tangible evidence of structural racism in our environment can help move us toward mitigation as we seek to provide resources for these families and improve equitable care.^21^ While there was no significant association between FI and preference for English or non-English language in our population, we were unable to analyze further by language preference due to the small numbers of families in each language group.

Patients and families screened in our study were more likely to have FI as the child’s age increased, which has not previously been reported. This could be due to age restrictions on many public food assistance programs, competing priorities and costs for older children, or the amount of food they need. Alternately, it could reflect differences in what brings patients to seek care in the ED at different ages. There was also a strong association with public insurance status and FI, which means many of the families identified with FI may also be eligible for food assistance programs.

There was no difference in FI based on history of chronic disease. We had postulated that the presence of chronic or complex illness history in a child may present additional financial stressors, as this has been reported in other settings,^22^–^24^ but we did not see an association when stratifying by PMCA. This means there was no difference in FI in our sample between children with no past medical history, those with some type of chronic disease, or those with complex chronic disease.

The overall prevalence of FI throughout the US and in ED settings is high. Our data were collected before the onset of the COVID-19 pandemic, but FI has been sharply increasing more recently with the rise of significant economic challenges. A recent analysis of the US Census Bureau Household Pulse Survey found that FI doubled in the general population and tripled in households with children as of June 2020.^25^ It appears that with this increase, regional variation and disparities by race and ethnicity persist.^16^ Given the high prevalence and the compounding effects of structural racism and poverty for different groups, we believe universal screening for FI with the provision of resources is crucial to providing high-quality care in the pediatric ED. With the sharp increase in economic vulnerability as a result of the COVID-19 pandemic, this need for universal screening in healthcare settings is even more crucial.

The HVS is a validated tool that can be rapidly completed and is recommended for screening as a part of a toolkit to address FI released by the American Academy of Pediatrics.^16^ Although it is commonly integrated in general pediatrician outpatient clinic visits as part of preventive care, it is not routinely implemented in most pediatric EDs. In previous research, families were more likely to report FI when completing written questions vs verbal.^26^ In our study, most families also preferred to answer written screening questions rather than verbally. The screening took an average of five minutes using the validated HVS tool, which makes it amenable to include in the routine ED check in process, particularly if self-administered by most families. Ideally responses should be entered directly into the electronic health record (EHR), with an electronic flag for providers when families identify as food insecure.

Despite the importance of FI screening and the availability of good screening tools, one challenging barrier to implementation is a process for connecting families with FI to food resources.^27^ In our study, our dedicated screener was also responsible for providing families who screened positive with food resources including local food banks, our hospital food pantry, and enrollment in nutritional assistance programs when eligible. The average time to present these resources to families was 10–15 minutes; the screener also spent time each week checking to make sure the resources were current. This more significant time investment requires planning by the ED team and consideration of who will be responsible for addressing families with FI when identified, and how this will integrate with other ED care. Given the critical role food plays in health, FI must be recognized as an important part of addressing the healthcare needs of the ED patient and should be achievable at some point during the ED visit. The research assistant for our study was neither a clinician or social worker. They became well-versed in available food resources and assisted and informed families of these. Thus, there are many creative personnel potential solutions for performing this role.

Our next step is the implementation of universal screening in written format with integration into the Electronic Health Record. We also hope to provide written materials in multiple languages that list locally available food resources. More research is needed on the ideal way to provide information to families with FI, but a connection to available resources after a positive screen is crucial.

## LIMITATIONS

This study has several limitations. There may have been a selection bias given our sampling method of convenience. We attempted to mitigate for this by ensuring screening was available and deployed during a representative variety of times of day and week. This study took place in a freestanding pediatric hospital, and we had a dedicated research assistant available to do the screening; this may limit generalizability to other centers. We only had written materials translated into Spanish and Somali. Families with another language preference did not have the option to answer questions in written format. Although we used video interpretation and did not exclude these families, the lack of translated materials may have limited the number of families we screened and/or limited the ability to provide resources for them. Finally, our numbers of families included who have differing language preferences was relatively low making it difficult to fully analyze the impact of language on risk for FI.

## CONCLUSION

Food insecurity was common among ED patients in our academic, freestanding pediatric ED, adding to a body of literature on the relatively high prevalence of FI in pediatric EDs. We found an association between FI and Black race, Hispanic ethnicity, public insurance, and increasing patient age. There were no significant associations between language preference and patient complexity. Using the Hunger Vital Sign tool, screening was feasible, and most caregivers preferred to complete the questions in written format when asked. Connecting families to food resources can be done by a variety of differing staff roles and will require additional time. Universal screening for FI with provision of food resources is feasible and necessary in pediatric EDs to provide optimal care for patients at highest risk for inequities and poor health outcomes.

## Supplementary Information



## Figures and Tables

**Figure:**
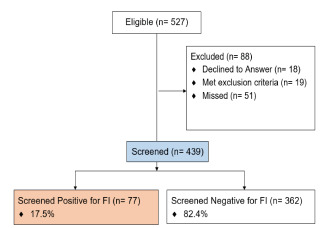
Study flow chart of patients screened using the Hunger Vital Screening Tool for food insecurity. *FI*, food insecurity.

**Table 1:** Characteristics of pediatric patients screened for food insecurity.

	All subjects (n=439)	Screened positive (n=77)	Screened negative (n=362)
Age, years, (IQR)	6.1 (2.2–11.8)	5.9 (2.1–11.8)	7.8 (2.9–14.2)
Gender N(%)			
Male	240 (54.7)	40 (52.0)	200 (55.3)
Female	199 (45.3)	37 (48.0)	162 (44.8)
Race/Ethnicity N(%)			
White	197 (46.5)	13 (46.5)	184 (52.7)
Hispanic	75 (17.7)	24 (32.0)	51 (14.6)
Asian	53 (12.5)	4 (5.3)	49 (14.0)
Black	51 (12.0)	20 (26.7)	31 (8.9)
Mixed or other	48 (11.3)	14 (18.7)	34 (9.7)
Preferred language N(%)			
English	374 (85.2)	54 (70.1)	320 (88.4)
Spanish	29 (6.6)	15 (19.5)	14 (3.9)
Somali	6 (1.4)	3 (3.9)	3 (0.8)
Other	30 (6.8)	5 (6.5)	25 (6.9)
Insurance N(%)			
Commercial	209 (47.6)	9 (11.7)	200 (55.3)
Medicaid	208 (47.4)	65 (84.4)	143 (39.5)
Uninsured	16 (3.6)	2 (2.6)	14 (3.9)
Military	6 (1.4)	1 (1.3)	5 (1.4)
Mental health N(%)	29 (6.6)	6 (7.9)	23 (6.4)
Length of visit, mean (SD)	3:51 (2:11)	3:51 (2:20)	3:51 (2:09)
Time of visit* N(%)			
Higher volume	277 (63.1)	43 (55.8)	234 (64.6)
Lower volume	162 (36.9)	34 (44.2)	128 (35.4)
PMCA N(%)			
Non-chronic	287 (65.4)	44 (57.1)	243 (67.1)
Non-CC	86 (19.6)	18 (23.45	68 (18.8)
Complex chronic	66 (15.0)	15 (19.5)	51 (14.1)
ESI, Med (IQR)	3.0 (2.0–3.0)	3.0 (3.0–4.0)	3.0 (2.0–3.0)
Disposition N(%)			
Discharged	347 (79.0)	63 (81.8)	234 (64.6)
Admitted	92 (21.0)	14 (18.2)	128 (35.4)

* Higher volume: between 2 PM – 2 AM, Lower volume: between 2 AM – 2 PM.

*IQR*, interquartile range; *SD*, standard deviation; *PMCA*, patient medical complexity algorithm; *non-CC*, non-complex, non-chronic; *ESI med*, Emergency Severity Index, median.

**Table 2:** Factors associated with positive food insecurity screening.

	OR	95% CI	P-value
Age, per year increase	1.05	1.01, 1.09	0.022
Gender			
Male	Ref	Ref	Ref
Female	1.26	0.71, 2.26	0.432
Race/Ethnicity			
Non-Hispanic White	Ref	Ref	Ref
Asian	0.84	0.24, 2.92	0.784
Black	5.21	2.13, 12.77	<0.001
Hispanic	3.47	1.48, 8.15	0.004
Mixed or other	3.81	1.54, 9.39	0.004
Insurance			
Commercial	Ref	Ref	Ref
Medicaid	5.74	2.52, 13.07	<0.001
Military	2.84	0.25, 32.06	0.399
Uninsured	1.47	0.26, 8.36	0.664
PMCA			
Non-chronic	Ref	Ref	Ref
Complex chronic	1.23	0.56, 2.67	0.606
Non-complex chronic	1.53	0.76, 3.11	0.237
Language			
English	Ref	Ref	Ref
Non-English	1.21	0.59, 2.48	0.594

*OR*, odds ratio; *CI*, confidence interval; *PMCA*, patient medical complexity algorithm.
